# An Improved Aerial Target Localization Method with a Single Vector Sensor

**DOI:** 10.3390/s17112619

**Published:** 2017-11-14

**Authors:** Anbang Zhao, Xuejie Bi, Juan Hui, Caigao Zeng, Lin Ma

**Affiliations:** 1 College of Underwater Acoustic Engineering, Harbin Engineering University, Harbin 150001, China; zhaoanbang@hrbeu.edu.cn (A.Z.); bixuejie@hrbeu.edu.cn (X.B.); cgzeng@hrbeu.edu.cn (C.Z.); malin@hrbeu.edu.cn (L.M.); 2 Science and Technology Underwater Acoustic Laboratory, Harbin Engineering University, Harbin 150001, China

**Keywords:** aerial target, height estimation, elevation, matching, compensation

## Abstract

This paper focuses on the problems encountered in the actual data processing with the use of the existing aerial target localization methods, analyzes the causes of the problems, and proposes an improved algorithm. Through the processing of the sea experiment data, it is found that the existing algorithms have higher requirements for the accuracy of the angle estimation. The improved algorithm reduces the requirements of the angle estimation accuracy and obtains the robust estimation results. The closest distance matching estimation algorithm and the horizontal distance estimation compensation algorithm are proposed. The smoothing effect of the data after being post-processed by using the forward and backward two-direction double-filtering method has been improved, thus the initial stage data can be filtered, so that the filtering results retain more useful information. In this paper, the aerial target height measurement methods are studied, the estimation results of the aerial target are given, so as to realize the three-dimensional localization of the aerial target and increase the understanding of the underwater platform to the aerial target, so that the underwater platform has better mobility and concealment.

## 1. Introduction

Underwater platforms have good concealment and mobility, have a greater self-sufficiency, endurance and combat radius, and can attack sea and land targets, and thus play an important role in national naval equipment. However, the self-defense ability of underwater platforms is poor. The effective observation and defense means against aerial targets are insufficient. The threat of aerial targets to underwater platforms can be fatal, so it is necessary to study how to detect and locate aerial targets with underwater sensors.

Before the study of the aerial target detection and localization algorithm, it is necessary to study the field excited by the aerial target first. There are four main ways an aerial target radiates noise: the direct refraction wave (namely the direct sound), the surface-seabed reflection wave, the non-uniform wave and the scattered wave. There are many studies on the field excited by the aerial target. Hudimac [1] studied the underwater field exited by aerial static targets and gave the sound intensity calculation formula of the aerial static target based on ray theory. Weinstein did anumerical calculation of the field exited by an aerial target based on the wave theory [2]. Brekhovskikh studied the reflection and refraction of the spherical wave at the flat interface and divided the acoustic waves reaching the underwater receiver into two parts: the underwater refraction waves and non-uniform waves according to ray theory. The non-uniform wave amplitude attenuated exponentially with the distance from the receiver to the interface [3]. Urick calculated the intensity of the direct refraction wave based on ray theory. There is a cosine square direction in the vertical direction of water [4]. Also based on wave theory, Chapman [5] gave the normal mode representation of the field excited by an aerial target. It is found that the excitation field of the aerial target can be equivalent to the underwater field, and the difference between the two is the excitation coefficient. The above studies are most based on the theoretical study of the underwater field excited by the aerial target. Buckingham verified that the aerial target radiated noise can be received underwater through several sea experiments [6], and the Doppler information of the received spectrum can be used in the aerial target’s recognition and localization. The sound propagation theory of the moving source in the three-layer Pekeris waveguide was deduced in detail. The energy and Doppler of the pressure field were analyzed in detail [7]. Clark and Jacyna [8,9] studied the effects of source motion on underwater received signals based on ray theory. Guthrie and Hawker [10,11] gave the field of the uniform linear motion source along the horizontal direction in the horizontal stratified medium based on normal mode theory. They obtained an approximate steady point calculation method, which made the realization of the normal mode method easier, but the method requires that the velocity of the source must be much smaller than the velocity of the medium. Schmidt [12] gave the field under the combined motion of the source and the receiver in the ocean waveguide environment with the use of the spectrum theory. This method is mainly applicable to long-distance Doppler pulse signal calculations. Ferguson [13] used the ray model to show how the Doppler frequency deviation of the received signal changes with time. The Doppler frequency offset of the line spectrum radiated by the aerial target, which is obtained by processing the experimental data, is in good agreement with the established ray model.

Through detecting the noise energy radiated by the aerial target, an underwater platform can detect and locate an aerial target. The aerial target radiated noise can be received by the sensors carried on the underwater platform. The phenomenon that the underwater sensor received signals consist of a large number of low frequency line spectra had been demonstrated by Urick [4], Medwin [14], and Reid [15]. Based on the line spectrum information included in the acoustic energy of the underwater received signal, the localization of the aerial target can be realized.

There are three methods to detect aerial targets through an underwater platform. The first method is direct observation or radar detection. Although the method is simple, the platform is easil exposed during the detection process, and the probability of being attacked by the aerial targets is therefore greatly increased. The second method is that the underwater platform releases buoys, and then uses sensors carried on these buoys for detection. This second method is affected by the environmental conditions, and constrained by the cable between the underwater platform and the buoy. Underwater platforms cannot dive to a greater depth, so invisibility is still not high. The third method is to use the sensors carried on the underwater platform to collect the signal for passive detection. Based on the Doppler characteristics of the aerial target radiated noise received by the underwater sensors, we can estimate the parameters of the aerial target. This method has high concealment and safety performance, and has no effect on the underwater platform’s mobility.

The previous studies on the field excited by the aerial target are mainly at the pressure field level, and mainly focus on field theory analysis. In this paper, we mainly study the field excited by the aerial target at the vector field level, and use the signals collected by a three-dimensional vector sensor to estimate the azimuth, frequency, heading angle, velocity, closest distance, horizontal distance, elevation and height. The target motion analysis (TMA) passive localization technique based on azimuth-Doppler frequency measurement is used to determine the target position. Since the frequency contains the Doppler shift due to the relative motion between the target and the receiver, it contains the state information of the moving target [16], so that the azimuth and frequency information can be used to carry out the estimation of various parameters. The localization of the aerial targets can be realized finally. The previous studies about the aerial target localization are applicable to the situation that the azimuth is not close to the heading angle. However, there exists the situation that the azimuth is close to the heading angle, such as the sea experiment data used in this paper. At this time, the parameter estimation results with the use of the previous existing methods are unsatisfactory. Aiming at the problems existing in the actual data processing, the motivation of this paper is to propose an improved algorithm to reduce the accuracy requirements of the angle estimation algorithm and to improve the robustness and precision of the parameters estimation. What’s more, we propose an elevation angle estimation algorithm so as to realize the estimation of the aerial target height and the three-dimension localization of the aerial target.

## 2. The Basic Principle of Passive Localization in the Horizontal Direction

### 2.1. Doppler Phenomenon in the Stratified Media

**Figure 1:**
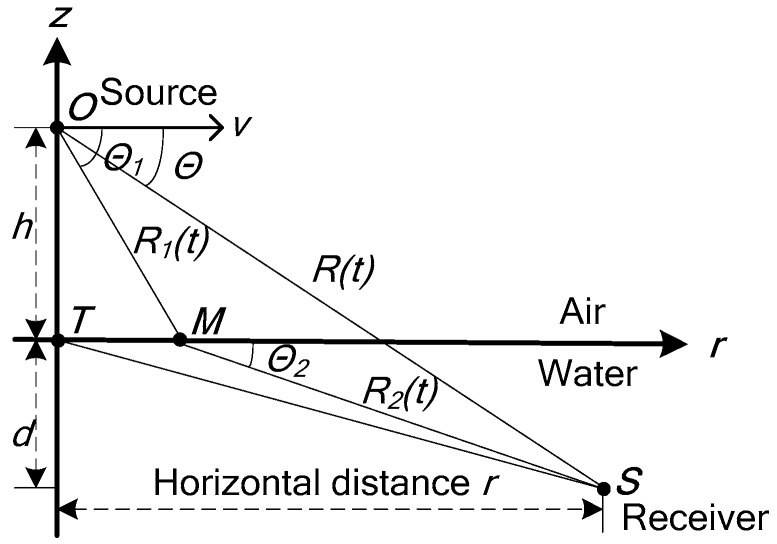
The direct refraction wave ray trace in the stratified medium.

A Doppler shift occurs when the source is in motion relative to the receiver. The Doppler frequency shift is related to the direction of the source movement, the signal frequency, the velocity of the source and the velocity of the medium. In Figure 1, the source is located at the point *O*, the underwater sensor (receiver) is located at the point *S*, the point *M* is the incident point of the direct refraction wave. *h* is the height of the source relative to the sea surface. *d* is the depth of the receiver relative to the sea surface. *T* is the point just below the source in the sea surface. *R*(*t*) is the distance between the source position *O* and the receiver position *S* in the horizontal direction, *R*_1_(*t*) is the distance between the source position *O* and the incident point *M* in the horizontal direction, *R*_2_(*t*) is the distance between the incident point *M* and the receiver position *S* in the horizontal direction.

Assuming that the stratified medium is uniform and stationary, the source moves in the horizontal direction, and the Doppler frequency of the received signal is [17]: $f_{R}=f_{0}*\frac{1}{1-M\cos\Theta }$, $f_{0}$ is the source frequency, M=v/c is the ratio of the source velocity *v* to the medium velocity *c*, Θ is the angle between the source-receiver direction and the source motion direction. Since c>v≥vcosΘ, then $\frac{v\cos\Theta }{c}<1$, ${\left(\frac{v\cos\Theta }{c}\right)}^{2}<<1$, therefore $f_{R}=f_{0}+\Delta f=f_{0}*\frac{1+\frac{v}{c}\cos\Theta }{\left(1-\frac{v}{c}\cos\Theta )(1+\frac{v}{c}\cos\Theta \right)}=f_{0}*(1+\frac{v}{c}\cos\Theta )$, $f_{R}$ represents the received Doppler frequency, Δf represents the Doppler frequency offset, $\Delta f=f_{0}*\frac{v}{c}\cos\Theta$.

In the horizontal stratified medium, according to the Snell theorem: $\frac{\cos\Theta_{1}}{c_{1}}=\frac{\cos\Theta_{2}}{c_{2}}$, $c_{1}$ is the sound velocity in the air, $c_{2}$ is the sound velocity in the water. $\Theta_{1}$ is the angle between the incident ray of the direct refraction wave and the source motion direction. $\Theta_{2}$ is the angle between the refraction ray of the direct refraction wave and the source motion direction. The received Doppler frequency $f_{R}$ does not change. That is, the incident wave in the air and the refraction wave in the water have the same Doppler frequency.

When the source is located in the air and the receiver is located in the water, the sound waves received by the sensor consist of two parts: normal mode and non-uniform wave. The non-uniform wave transmits energy to the underwater receiver in a manner different from the refraction wave, as shown in Figure 1. The normal mode arrives at the receiving point *S* along the propagation path *OTS* and the path *OTMS*, while the non-uniform wave travels along the path *OMS* to the receiving point.

Path *OMS*: It is the transmission path of the normal mode in the air. This kind of wave is surface wave (non-uniform wave) below the air-water interface, its intensity decreases exponentially with the depth. This kind of wave is strong in the short-range. Its velocity signal has larger energy than its pressure signal, and has larger line spectrum Doppler shift. The Doppler frequency offset $\Delta f_{1}$ is given by the following expression [18]:

(1) $$\Delta f_{1}=f_{0}\frac{v}{c_{1}}\cos\varphi \cos\alpha$$

φ is the horizontal angle between the source motion direction and the sound propagation direction, α is the elevation angle.

Path *OTMS* and *OTS*: Path *OTMS* is the transmission path of the normal mode whose eigenvalues are complex in the water and its Doppler frequency offset is $\Delta f_{2}$. The normal mode transmitting along the path *OTS* in the water, is standing wave in the vertical direction of water, $\Delta f_{3}$ is its Doppler frequency offset:

(2) $$\Delta f_{2}=f_{0}\frac{v}{c_{2}}\cos\varphi ,\;\Delta f_{3}=f_{0}\frac{v}{c_{2}}\cos\varphi$$

The instantaneous frequencies of the line spectrum signals arriving along the path *OMS* and *OTMS* are $f_{1}$ and $f_{2}$, respectively [18]:

(3) $$f_{1}(t)=f_{0}+\Delta f_{1},\;f_{2}(t)=f_{0}+\Delta f_{2}$$

The Doppler frequency offsets of the signals arrive along the *OTMS* and *OTS* paths are smaller, and their signal intensities are weak at the close range, so that during the frequency estimation process, the spectrum extraction of signals transmitting along the *OTMS* and *OTS* paths are more complex and more difficult, so in this paper, the line spectrum signal transmitting along the *OMS* path is the main research object.

### 2.2. Angle Estimation Method

The field contains pressure and velocity information, the former is the scalar field, while the latter is the vector field. Single point pressure is omnidirectional. For the traveling wave, the velocity signal matches the direction of arrival. The vector sensor is a kind of acoustic sensor used to measure the underwater vector field, which is made up of pressure sensor and velocity sensor (pressure gradient sensor, accelerometer, displacement meter, etc.) [19]. The vector sensor outputs the pressure and the orthogonal three-dimensional (two-dimensional) velocity components [20].

The three orthogonal components of the velocity *v* are:

(4) $$\{\begin{array}{l} v_{x}=v(t)\cos\theta \cos\alpha \\ v_{y}=v(t)\sin\theta \cos\alpha \\ v_{z}=v(t)\sin\alpha \end{array}$$

v(t) is the velocity waveform. θ is the horizontal azimuth angle (range: 0–360°), the x-axis positive direction is 0°, α is the elevation angle (range: −90–90°), the horizontal plane (*xoy* plane) is 0°.

**Figure 2:**
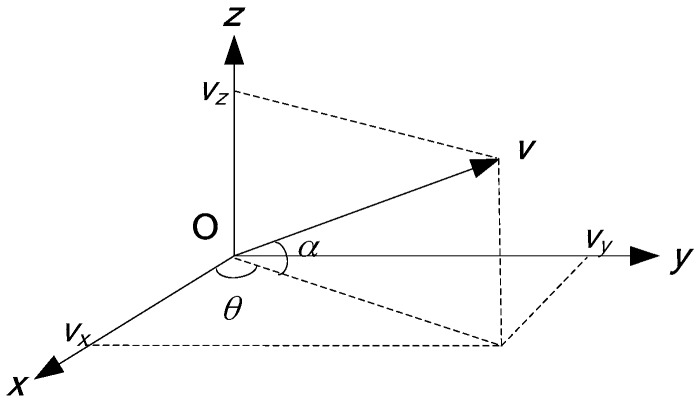
The projection of velocity *v* and its three orthogonal components $v_{x},v_{y},v_{z}$, considering θ,α.

Figure 2 illustrates the geometric relationship of the projection. If the pressure p(t) meets p(t)=x(t), the field is assumed to meet the Ohm law, then $v(t)=\frac{1}{\rho c}x(t)$. x(t) is the target signal. According to (4), we can get:

(5) $$\{\begin{array}{l} v_{x}(t)=\frac{1}{\rho c}x(t)\cos\theta \cos\alpha \\ v_{y}(t)=\frac{1}{\rho c}x(t)\sin\theta \cos\alpha \\ v_{z}(t)=\frac{1}{\rho c}x(t)\sin\alpha \end{array}$$

In the discussion of signal processing problems, we can omit the acoustic impedance ρc in the (5) and set ρc=1.

The signal received by the underwater sensor is the refraction signal of the noise radiated by the aerial target. The expression of the received refraction signal is:

(6) $$\left\{ \begin{array}{l} y_{p}(t)=W\cdot x(t)+n_{p}(t)\\ y_{v_{x}}(t)=W\cdot x(t)\cos\theta \cos\alpha +n_{vx}(t)\\ y_{v_{y}}(t)=W\cdot x(t)\sin\theta \cos\alpha +n_{vy}(t)\\ y_{v_{z}}(t)=W\cdot x(t)\sin\alpha +n_{vz}(t) \end{array}\right.$$

$n_{p}(t)$, $n_{vx}(t)$, $n_{vy}(t)$ and $n_{vz}(t)$ are the isotropic noise components of pressure and velocity received by the vector sensor, and they are all independent of x(t). *W* is known as the refraction coefficient. $W=\frac{2q\sin\Theta_{1}}{q\sin\Theta_{1}+\sin\Theta_{2}}$, $q=\frac{\rho_{2}c_{2}}{\rho_{1}c_{1}}$. $\rho_{1}$ is the air density, $\rho_{2}$ is the water density.

The physical basis of the complex acoustic intensity’s anti-interference performance is the correlation between the target pressure signal and the target velocity signal, whereas the pressure and velocity of isotropic environment interference is irrelevant or weakly correlated. The average output of the complex acoustic intensity is:

(7) $$\langle I_{x}(f)\rangle =W^{2}\langle y_{P}(f)y_{V_{x}}^{*}(f)\rangle =W^{2}\langle {\left|X(f)\right|}^{2}\rangle \cos\theta \cos\alpha +\delta (f)$$

(8) $$\langle I_{y}(f)\rangle =W^{2}\langle y_{P}(f)y_{V_{y}}^{*}(f)\rangle =W^{2}\langle {\left|X(f)\right|}^{2}\rangle \sin\theta \cos\alpha +\delta (f)$$

〈⋅〉 represents sliding-average periodgram operation. “_*_” represents complex conjugate, |⋅| represents modulus operation, and $y_{P}(f)$, $y_{V_{x}}(f)$, $y_{V_{y}}(f)$ are the Fourier transform of the pressure and the velocity respectively, X(f) is the Fourier transform of the signal, δ(f) is the Fourier transform of the interfere with the small quantity.

In the underwater acoustic channel, the acoustic Ohm law is approximately satisfied. Therefore, the pressure signal has the same phase as the velocity signal. According to the basic properties of the Fourier transform, the energy of the two signals in the same phase is concentrated on the real component of the cross spectrum. The imaginary component of the cross spectrum only contains the energy of interference and noise [20,21].

The cross spectrum method at single frequency point is given by:

(9) θ(f)=tan−1(Re[〈Iy(f)〉]Re[〈Ix(f)〉])

Re[⋅] represents the operation of getting the real component.

In this paper, we use the weighted bar graph method to do the statistical analysis of the azimuth estimation results. The specific algorithm of the weighted bar graph method is given by:

(10) $$\zeta_{k}=\{\mathrm{mod}[\theta (f_{k}),2\pi ]\cdot 180/\pi \},(1\leq k\leq M)$$

(11) $$A(f_{k})=\sqrt{I_{x}^{2}(f_{k})+I_{y}^{2}(f_{k})}$$

(12) $$Ζ(n)=\sum_{k}A(f_{k})$$

ζ is the azimuth sequence in the angle domain, {⋅} represents the getting integer operation towards positive infinity, mod[⋅] represents the modulus operation, $\theta (f_{k})$ is the azimuth estimation value at every frequency point, *k* is the sequence number of the frequency point, *M* is the total number of frequency points used in the azimuth estimation. $A(f_{k})$ is the complex acoustic intensity of the *k*-th frequency point. *Z* is an array whose dimension is 1 × 360. We use the array *Z* to do the weighted statistic of the azimuth sequence ζ. The complex acoustic intensity $A(f_{k})$, used for summation in (12), meets $\zeta_{k}=n(n=1,2,\cdots 360)$. *n* is an angle sequence which varies from 1° to 360°.

Using the azimuth estimation method based on (9), we can calculate the azimuth value at every frequency point. Then we use the weighted bar graph method to do a statistical analysis of the azimuth estimation result at every frequency point, so as to get the probability distribution of the azimuth estimation at a certain time. The azimuth corresponding to the maximum value of the probability distribution is the desired target azimuth.

In this section, we introduce the angle estimation method with a single vector sensor in the horizontal direction, namely, we give the estimation method of the azimuth angle. The angle estimation method in the vertical direction (elevation angle) is introduced in the Section 3.1.

### 2.3. Frequency Estimation Method

In this paper, we use the harmonic cluster adaptive line spectrum enhancer to extract the aerial target base frequency [22]. Assuming that the aerial target signal consists of harmonic cluster line spectrum whose shaft frequency is $f_{0}$, the modulation line spectrum cluster is:

(13) $$p(t)=\sum_{n=1}^{N}\cos(2\pi nf_{0}t)+n(t)$$

$f_{0}$ is the shaft frequency, there are *N* harmonic components; *n* takes from the natural number between 1 and *N*; *t* represents the time; *n*(*t*) represents the background noise signal.

**Figure 3:**
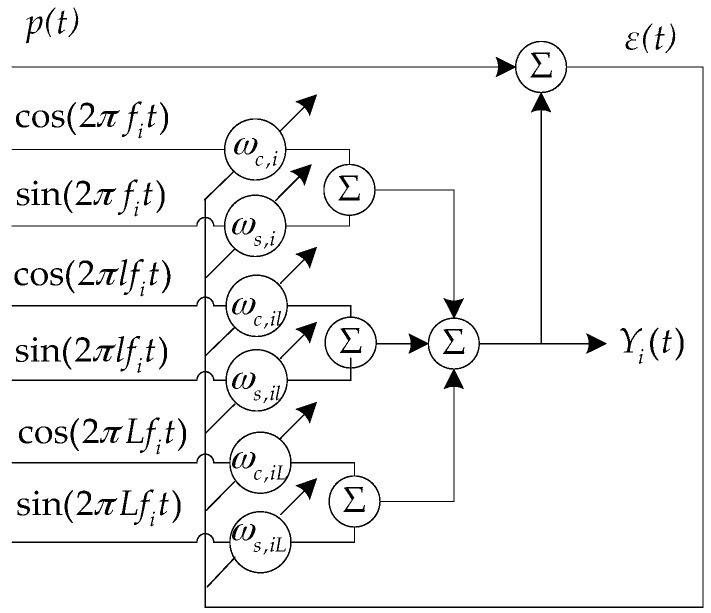
The principle of harmonic cluster adaptive line spectrum enhancer.

Figure 3 shows the structure of the harmonic cluster adaptive line spectrum enhancer. By frequency sweeping, the harmonic component of the input signal can be enhanced when the harmonic component of the input signal *p*(*t*) corresponds to the frequency of the harmonic cluster reference input signal. The input signal is *p*(*t*), the shaft frequency of reference signal is $f_{i}$, the scanning step is Δf. We start scanning from the low frequency. Each scanned harmonic cluster has *L* sets of reference input signals. Two reference signals of the each set of input signals are mutually orthogonal. Respectively, they are:

(14) $$\{\begin{array}{c} r_{s_{i}}=\cos(2\pi lf_{i}t)\\ r_{c_{i}}=\sin(2\pi lf_{i}t) \end{array}$$

the center frequency of each set of reference signals is harmonically related. *l* takes the natural number between 1 and *L*. Adjust the base frequency $f_{i}$ of the harmonic cluster. The range of $f_{i}$ is the frequency range of the shaft frequency. During each scanning of the base frequency, the harmonic cluster is accumulated, the iterative formula of the weight vector is:

(15) $$Y_{i}(k)=\sum_{l=1}^{L}w_{c,il}\cdot \cos(2\pi lf_{i}k/f_{s})+\sum_{l=1}^{L}w_{s,il}\cdot \sin(2\pi lf_{i}k/f_{s})$$

(16) $$\varepsilon_{i}(k)=p(k)-Y_{i}(k)$$

(17) $$\{\begin{array}{c} W_{c}(k+1)=W_{c}(k)+\mu \varepsilon_{i}(k)r_{c}(k)\\ W_{s}(k+1)=W_{s}(k)+\mu \varepsilon_{i}(k)r_{s}(k) \end{array}$$

$Y_{i}$ is the harmonic cluster adaptive line enhancer (ALE) output at the base frequency; $W_{c}$ and $W_{s}$ are the *L* × 1 order weight vectors whose initial values are both 0. $r_{c}=\{r_{c_{i}}(k)\},r_{s}=\{r_{s_{i}}(k)\},i=1,2,\cdots ,L$. μ is a constant parameter whose value is 0.001. When the base frequency $f_{i}$ of the harmonic cluster or its harmonic frequencies do not coincide with the line spectrum, the frequency spectrums at the frequency of $f_{i}$ and its harmonic frequencies are not enhanced, the noise is not enhanced too. After the sweep processing in the whole frequency band, when $f_{i}=f_{0}$, the system output line spectrum energy is the largest, through the spectrum level signal noise ratio (*SNR*) maximum criterion, the harmonic cluster adaptive line spectrum enhancer’s best output can be selected. At this time, the reference base frequency $f_{i}$ is the shaft frequency of the target.

### 2.4. Geometric Relationship of Passive Localization in the Horizontal Direction

**Figure 4:**
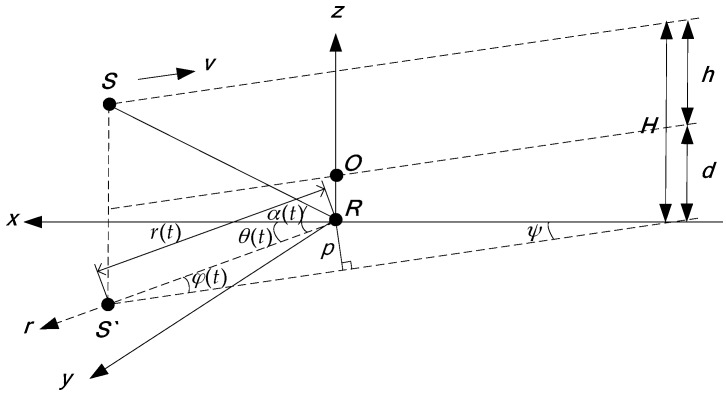
The passive localization geometry schematic diagram.

Assuming that the aerial target, located at the point *S*, is flying at a constant altitude *h* (the reference surface being the sea surface), and moving on a straight line at the constant velocity v. The depth of the underwater sensor, located at the point *R*, is d (the reference surface: the sea surface). The projection of *S* at the *xoy* surface is $S^{\prime }$. The point *R* is considered as the origin of the Cartesian coordinate system. *O* is the point directly above the receiving sensor in the sea surface. *H* is the vertical distance between the source and the receiving sensor. The passive localization geometry relationship is shown in Figure 4. In this paper, all of the following horizontal distances are the distances between the source and the receiving sensor in the horizontal direction, and all of the following vertical distances are the distances between the source and the receiving sensor in the vertical direction. r(t) is the target horizontal distance, *p* is the closest horizontal distance, ψ is the angle between the motion path and the x-axis positive direction, and θ(t) is the target horizontal azimuth angle. φ(t) is the angle between the source motion direction and the sound propagation direction. α(t) is the target elevation angle in the vertical direction. Taking any reference time *t* = 0, the position at this time is $\left(x_{0},y_{0}\right)$, the target azimuth is $\theta_{0}=\theta (0)$. The horizontal distance is $r_{0}=\sqrt{x_{0}^{2}+y_{0}^{2}}=r(0)$.

The motion equations in the *xoy* plane are: $x(t)=v_{x}t+x_{0},y(t)=v_{y}t+y_{0}$, $v_{x}$ and $v_{y}$ are the two orthogonal components of the velocity v, $v_{x}=v\cos\psi$, $v_{y}=v\sin\psi$, x(t)=r(t)cosθ(t), y(t)=r(t)sinθ(t), $\cos\theta (t)=\frac{x(t)}{r(t)}=\frac{x(t)}{\sqrt{x^{2}(t)+y^{2}(t)}}$, $\cos\alpha (t)=\frac{r(t)}{\sqrt{r^{2}(t)+H^{2}}}$, H=h+d. In polar coordinates, the straight line equation is $r(t)=\frac{p}{\sin\varphi (t)}=\frac{p}{\sin[\theta (t)-\psi ]}$. Therefore, it can be seen from the above formulas that the passive localization can be realized after obtaining the values of p,θ(t),ψ in the actual measurement [23].

In the geometrical schematic diagram of passive localization shown in Figure 4, the position of the receiver is the origin of the coordinate, and in the direct refraction wave ray trace shown in Figure 1, its coordinate origin is the incident point of the refraction wave. When the direct refraction wave is used as the main research signal of the passive localization, the direct refraction wave ray trace (*OMS*) is approximated to the line from the source to the receiver in this paper. The ray trace of the direct refraction wave shown in Figure 1 can be simplified to the ray trace in the passive geometrical schematic diagram shown in Figure 4. It can be seen from the geometrical relationship of Figure 1 and Figure 4 that, in the horizontal direction, the horizontal component of R(t) is $R_{xy}(t)=r(t)=R_{1}(t)\cos\Theta_{1}+R_{2}(t)\cos\Theta_{2}$, that is, the approximation does not change the magnitude of the horizontal distance and does not affect the estimation accuracy of the horizontal distance. It can also be seen that, in the vertical direction, the vertical component of R(t) is $R_{z}(t)=h+d=R_{1}(t)\sin\Theta_{1}+R_{2}(t)\sin\Theta_{2}$, namely, the approximation does not change the magnitude of the vertical distance and does not affect the estimation accuracy of the vertical distance. The relationship between Θ and α, φ is cosΘ=cosαcosφ.

### 2.5. The Basic Principle of the Conventional Parameter Estimation Algorithm

#### 2.5.1. Theoretical Foundation

In this paper, we use the signal arriving along the *OMS* path to estimate the parameters. The improved algorithm is proposed based on the conventional algorithm [23].

Only using the $f_{1}(t)$ data, when the aerial target operating conditions remain constant and the target radiates a series of line spectrums $f_{0n}$, $f_{0n}=nf_{01},n=1,2,3\ldots N$. *n* is the order number of the line spectrums, *N* is the total number of the line spectrums. Then the received signal frequency is $f_{0n}^{'}=n(1+\delta )f_{01}$, $\delta =\frac{v}{c_{1}}\cos\varphi \cos\alpha$. The estimation formula of the base frequency sequence $f_{01}$ is:

(18) $${\hat{f}}_{01}=\frac{\cos(\theta (t_{2})-\psi )\cos(\theta (t_{1})-\psi )}{\cos(\theta (t_{1})-\psi )-\cos(\theta (t_{2})-\psi )}[\frac{f_{0m}^{'}(t_{2})-f_{0n}^{'}(t_{2})}{\left(m-n)\cos(\theta (t_{2})-\psi \right)}-\frac{f_{0m}^{'}(t_{1})-f_{0n}^{'}(t_{1})}{\left(m-n)\cos(\theta (t_{1})-\psi \right)}]$$

*m* and *n* are line spectrum order numbers, *m* > *n* (*m* and *n*: positive integers).

The estimation formula of the heading angle ψ is:

(19) $$tg\hat{\psi }=\frac{t_{2}\sin(\theta_{0}-\theta (t_{1}))\sin\theta (t_{2})-t_{1}\sin(\theta_{0}-\theta (t_{2}))\sin\theta (t_{1})}{t_{2}\sin(\theta_{0}-\theta (t_{1}))\cos\theta (t_{2})-t_{1}\sin(\theta_{0}-\theta (t_{2}))\cos\theta (t_{1})}$$

The estimation formula of the velocity *v* is:

(20) $$\hat{v}=\frac{(f_{01}(t)-{\hat{f}}_{01})c_{1}}{{\hat{f}}_{01}\cos\alpha \cos(\theta (t)-\psi )}$$

**Figure 5:**
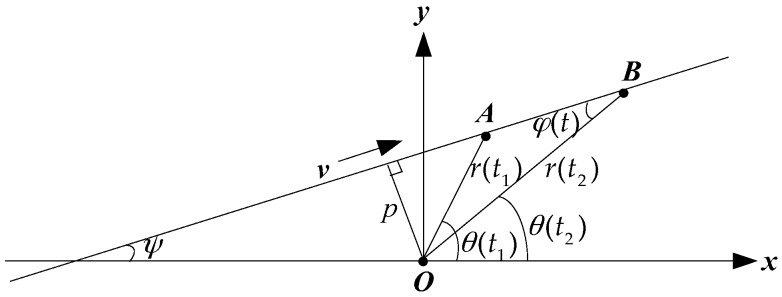
The track geometry diagram of the aerial target in the horizontal plane.

The projection of the passive localization geometry relationship in the *xoy* plane is shown in Figure 5. The projection of the target motion track in the *xoy* plane is the straight line where the points *A* and *B* are located on. 

According to the geometric relationship shown in Figure 5, we get the estimation formula of the closest horizontal distance *p*:

(21) $$\hat{p}=\frac{v(t_{2}-t_{1})\sin\varphi (t_{2})\sin\varphi (t_{1})}{\sqrt{\sin^{2}\varphi (t_{1})+\sin^{2}\varphi (t_{2})-2\sin\varphi (t_{2})\sin\varphi (t_{1})\cos(\theta (t_{1})-\theta (t_{2}))}}$$

The estimation formula of the target horizontal distance r(t) is:

(22) $$\hat{r}(t)=\frac{\hat{p}}{\sin(\theta (t)-\psi )}$$

#### 2.5.2. Simulation Results

The simulation parameters are shown in Table 1.

The initial distance $r_{0}$ is the horizontal distance at the initial time. The parameter *SNR* refers to the signal-to-noise ratio of the signal transmitted through the *OMS* path. Integration time length refers to the time length of the spectrum analysis. The range of the working frequency band is 10–250 Hz.

**Figure 6:**
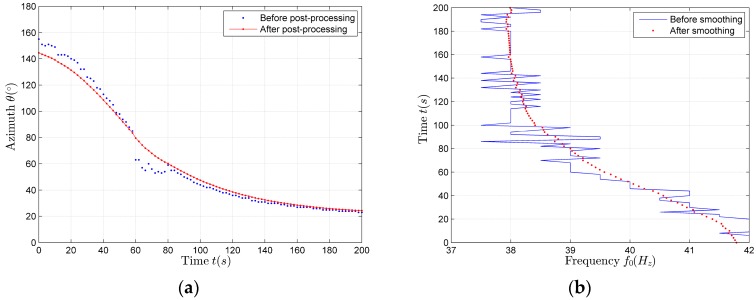
The estimation results of the simulation data. (**a**) Azimuth estimation results; (**b**) Source base frequency estimation results.

Using (9)–(12), we can estimate the target azimuth angle. We use the FFT spectrum of the target signal to estimate the source base frequency. We smooth the estimation results with the forward double α filtering method which is described in detail in the Section 4.3.2 . The estimation results are shown in Figure 6a,b.

Based on the azimuth and frequency estimation results shown in Figure 6a,b, we use the bar graph method to do the statistical analysis of the aerial target motion parameters (heading angle ψ, source base frequency $f_{01}$, velocity *v* and closest distance *p*).

**Figure 7:**
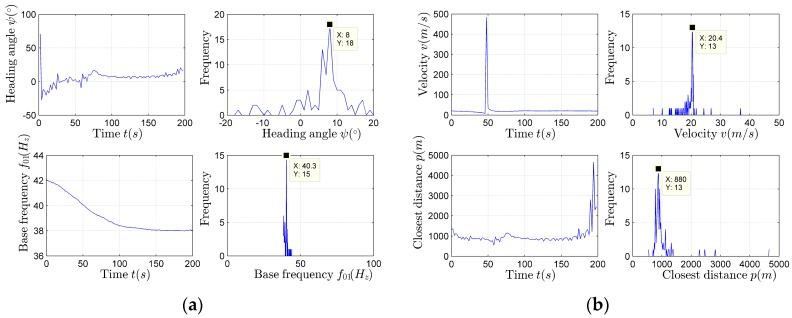
The distribution diagram and statistical histogram of the aerial target motion parameters in the horizontal direction. (**a**) The estimation results of the heading angle and the base frequency; (**b**) The estimation results of the velocity and the closest distance.

The statistical analysis results of the aerial target motion parameters are presented as the histogram in the paper, as shown in Figure 7a,b. According to Figure 7a,b, the parameter estimation values corresponding to the maximum in each histogram are the desired parameter estimation results. The parameter estimation results are shown in Table 2.

**Figure 8:**
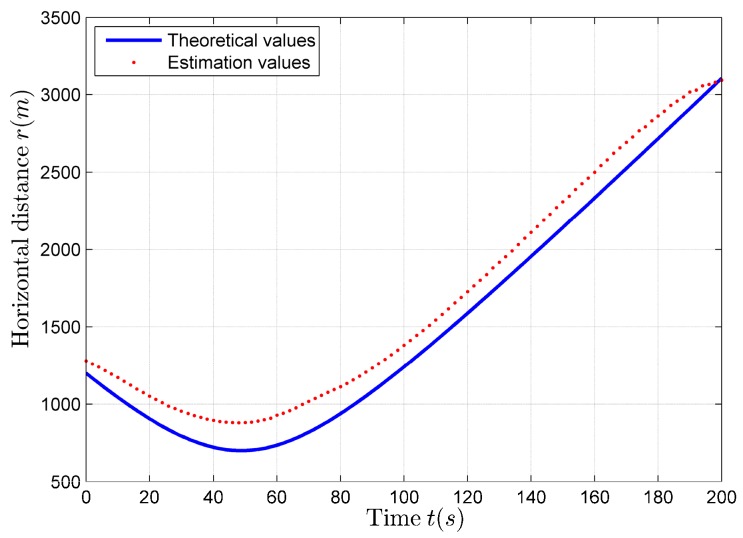
The comparison between the theoretical values and the estimation values of the horizontal distance.

According to the parameter estimation results shown in Table 2, using (22), we can obtain the horizontal distance estimation results. The comparison between the theoretical values and the estimation values of the horizontal distance is shown in Figure 8.

### 2.6. The Basic Principle of the Improved Parameter Estimation Algorithm

The simulation results of the algorithm shown in Section 2.5 are very good, but in the practical application, the algorithm cannot achieve the effect in the simulation because of the limited azimuth and elevation estimation accuracy, so the following improvements are made to the algorithm.

#### 2.6.1. Improvement of the Frequency Estimation Algorithm

If the frequency estimation is made using (18), the estimation effect is poor when the azimuth changes slowly, namely $\cos[\theta (t_{1})-\psi ]\approx \cos[\theta (t_{2})-\psi ]$, and many maxima are estimated because there is a phenomenon that the denominator is approximately zero. The algorithm is improved by using the least squares method to fit the extracted spectrum sequence $f_{tn}$, so as to obtain the fitting result $f_{LSMn}$. Select the appropriate time period to estimate the base frequency. Firstly, the selected time period should satisfy that the frequency, which is almost constant, cannot account for a large proportion, otherwise the estimation result is that stable value. However, the stable value is generated when $\varphi \to 0^{\circ },\alpha \to 0^{\circ }$, at this time $f_{t}\approx f_{0}(1\pm \frac{v}{c})$, the base frequency estimation result $f_{t}$ is not the source frequency $f_{0}$ which is of great need. According to the time distribution curve, apart from meeting the above conditions, the selected time period for the base frequency estimation should be away from 0 and stable:

(23) $${\hat{f}}_{02}=\sum_{i=1}^{N}f_{LSMi}(t)$$

Compared with using ${\hat{f}}_{01}$ for subsequent parameter estimation, using ${\hat{f}}_{02}$ for subsequent parameter estimation has a better estimation effect.

#### 2.6.2. Improvement of Closest Distance Estimation Algorithm

In the estimation process of the closest horizontal distance *p*, there is also a problem that the estimation effect is poor when the azimuth changes slowly, because $\varphi (t)=\theta (t)-\psi ,\theta (t_{1})\approx \theta (t_{2}),\varphi (t_{1})\approx \varphi (t_{2})$, then $\sin(\varphi (t_{1}))\approx \sin(\varphi (t_{2})),\cos(\theta (t_{1})-\theta (t_{2}))\approx 1$, so the denominator of (21) is approximately zero, and there are a large number of maxima in the estimation results.

In this case, the estimation results obtained by using (21) cannot achieve the expected accuracy, so the algorithm should be modified as follows: in the time period when the horizontal distance is close to the closest distance, the following approximation is used:

(24) $$r^{2}(t_{2})=r^{2}(t_{1})+v^{2}{\left(t_{2}-t_{1}\right)}^{2}$$

Since $\theta (t_{1})\approx \theta (t_{2})$, $r(t_{1})\approx r(t_{2})$, thus in the triangle *OAB* shown in the Figure 5, $∠AOB=\theta (t_{1})-\theta (t_{2})\approx 0^{\circ }$, $∠OAB=∠OBA\approx 90^{\circ }$.

In this case, we use the matching algorithm to calculate the requested *p* value. The concrete steps of the matching algorithm are the following:

1. Assume a $\hat{p}$ value and obtain the corresponding $\hat{r}(t)$ estimation curve according to (22). Because the magnitude of the $\hat{p}$ value only affects the amplitude of $\hat{r}(t)$, $\hat{p}$ value does not affect the variation trend of $\hat{r}(t)$.
2. According to the $\hat{r}(t)$ curve, the more stable time period can be determined.
3. Through matching estimation at the selected $\hat{p}$ interval, scanning each $\hat{p}$ value, the corresponding velocity estimation sequence can be obtained.
4. The mean value of the velocity estimation results in the stable time period is taken as the estimated $\hat{v}$ value corresponding to the scanned $\hat{p}$ value.
5. When the $\hat{v}$ value is closest to the velocity estimation result obtained through using (20), the corresponding $\hat{p}$ is regarded as the optimal estimation result of the closest distance.

After obtaining the optimal estimation result of $\hat{p}$, according to (22), the $\hat{r}(t)$ estimation is still found to be discontinuous, and there are still quite a lot of maxima. Since the estimation is inaccurate and discontinuous, using the data at the previously determined stable time period as the initial data, after compensating the subsequent data, we can obtain more continuous and better estimation results.

#### 2.6.3. Horizontal Distance Compensation Algorithm

$\hat{r}(t)$ in the steady time period is used to compensate the subsequent estimation results. The appropriate time point $t_{1}$, in the steady time period, is used as the starting time. The compensation result of $\hat{r}(t_{2})$ is calculated as follows:

(25) $$\hat{r}(t_{2})=\sqrt{{\left(r(t_{1})\cos\theta (t_{1})+v(t_{2}-t_{1})\cos\psi \right)}^{2}+{\left(r(t_{1})\sin\theta (t_{1})+v(t_{2}-t_{1})\sin\psi \right)}^{2}}$$

Using the above parameter estimation formulas, as long as we estimate the azimuth sequence and frequency sequence, we can carry out the corresponding parameter estimation.

## 3. The Basic Principle of Passive Localization in the Vertical Direction

### 3.1. Elevation Estimation

#### 3.1.1. Vertical Sound Intensity Flow Method

According to (6) we can get the expression of the vertical sound intensity $I_{z}(f)$:

(26) $$\langle I_{z}(f)\rangle =W^{2}\langle y_{P}(f)y_{V_{z}}^{*}(f)\rangle =W^{2}\langle {\left|X(f)\right|}^{2}\rangle \sin\alpha +\delta (f)$$

Using the vertical sound intensity, we can estimate the elevation. According to (7), (8) and (26), the calculation formula of the elevation is obtained [24]:

(27) $$I_{h}(f)=\left[\begin{array}{c} I_{x}(f)\\ I_{y}(f) \end{array}\right]$$

(28) α=tan−1(Re[Iz(f)]|Re[Ih(f)]|)

#### 3.1.2. Frequency Sequence Extraction Method

According to (1), we can get another method to calculate the elevation:

(29) $$\alpha =\cos^{-1}\left[\frac{(f-f_{0})c_{1}}{f_{0}v\cos\varphi }\right]$$

### 3.2. Source Height Estimation

The receiver depth is assumed to be known in this algorithm.

#### 3.2.1. Estimation Algorithm 1

Use the above two methods to estimate the elevation α. According to the geometric relationship described in Section 2.4, it can be launched:

(30) $$H=\sqrt{\frac{r^{2}(t)}{\cos^{2}\alpha }-r^{2}(t)}$$

Due to the estimation of the horizontal distance r(t) has been done in the above section, the estimation result is $\hat{r}(t)$. The estimation formula of the aerial target height *h* can be obtained:

(31) $$h=H-d=\sqrt{\frac{{\hat{r}}^{2}(t)}{\cos^{2}\alpha }-{\hat{r}}^{2}(t)}-d$$

#### 3.2.2. Estimation Algorithm 2

**Figure 9:**
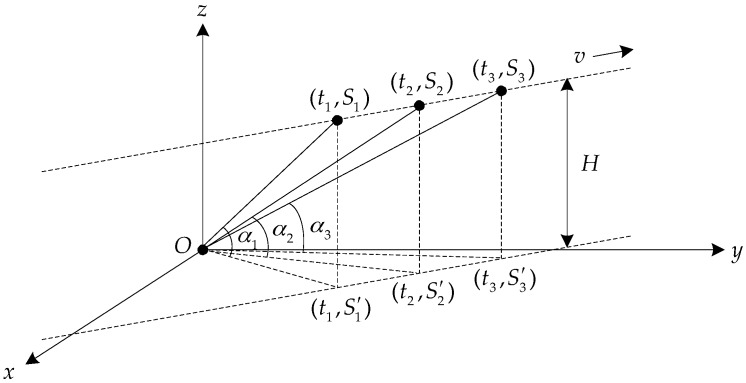
The motion track of the target moving on a straight line at the constant height and at the constant velocity.

The motion track of the aerial target is shown in Figure 9. The target is flying on a straight line at the constant height *H* (relative to the horizontal plane where the receiver is) and at the constant velocity *v*. The observation platform is located at point *O*. $S_{1}$, $S_{2}$ and $S_{3}$ are three source positions equally spaced in time. $t_{1}$, $t_{2}$ and $t_{3}$ are the observation time points of $S_{1}$, $S_{2}$ and $S_{3}$. $S_{1}^{\prime }$, $S_{2}^{\prime }$ and $S_{3}^{\prime }$ are the projection points of $S_{1}$, $S_{2}$ and $S_{3}$ in the *xoy* plane where the point *O* is. According to the Stewart’s theorem [25], the relationship between height *H* and the distance from the target projection point to the observation platform ($OS_{1}^{\prime },OS_{2}^{\prime },OS_{3}^{\prime }$) can be obtained:

(32) $$\frac{2v^{2}T_{0}^{2}}{H^{2}}=\cot^{2}\alpha_{1}-2\cot^{2}\alpha_{2}+\cot^{2}\alpha_{3}$$

(33) $$t_{2}-t_{1}=t_{3}-t_{2}=T_{0}$$

*T*_0_ is the observation time interval, and $\alpha_{1},\alpha_{2},\alpha_{3}$ are the elevation values observed at equal time intervals respectively.

The aerial target height (*h*, relative to the sea surface) estimation, based on (32) is given by:

(34) $$h=H-d=\sqrt{\frac{2v^{2}T^{2}}{\cot^{2}\alpha_{1}-2\cot^{2}\alpha_{2}+\cot^{2}\alpha_{3}}}-d$$

## 4. Sea Experiment Data and Results

On 7 January 2009, an aerial target noise measurement test was conducted in a certain area of Qingdao, China. The main purpose of this experiment was to obtain the aerial target’s underwater vector field data, and study the practical feasibility of using underwater sensors to locate aerial targets.

The experiment used a piezoelectric ceramic vector sensor, namely an accelerometer, to collect signals. The relationship between acceleration and velocity is:

(35) $$\vec{a}=j\omega \vec{v},\vec{v}=-j\frac{1}{\omega }\vec{a}$$

According to (35), we can get that the phase difference of the collected velocity and pressure information is 90 degrees. Before the subsequent calculation, the collected signals must be converted, and the collected acceleration signals are converted to the velocity signals.

**Figure 10:**
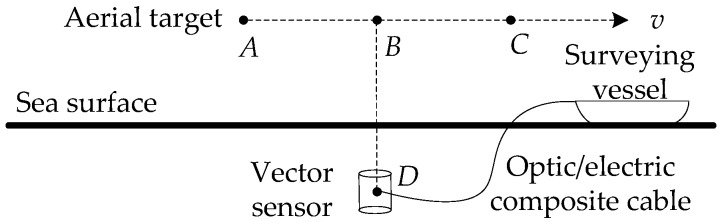
The profile of the sea experiment layout.

The test layout is shown in Figure 10. The three-dimensional low-frequency vector sensor is suspended underwater and connected to the surveying vessel by the optic/electric composite cable.

The test condition is that the sea depth is 50 m, the receiver depth is 25 m, and the target velocity is 80 km/h. The target appeared roughly after 220 s, at some point in the vicinity of the top of the sensor, and then away. The vector sensor was at point *D*. The aerial target ran from point *A* to point *C*, and flied at a constant velocity *v*. After the aerial target was in place, it flew over the vector sensor mounted on the underwater platform and then went away.

### 4.1. Time Domain Waveform and Frequency Domain Spectrum

The frequency estimation and the azimuth estimation of the data collected by the vector sensor are carried out after the time domain pre-whitening process, so as to reduce the influence of the background noise on the parameter estimation.

**Figure 11:**
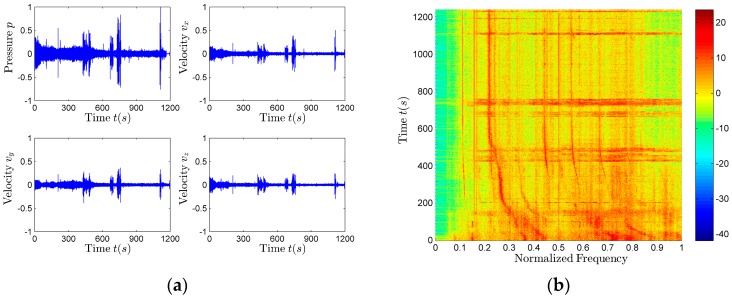
The time and frequency domain information of the signal. (**a**) The signal waveform; (**b**) The normalized spectrogram of $v_{z}$ signal.

The calculation formula of time domain pre-whitening method is:

(36) y(k)=x(k+m)−x(k)

among them, k=0,1,2,⋯ is the sample order numbers; *m* is the sampling number in the interval, we usually take 1–2 sampling interval. In this paper, *m* = 1, *x*(*k*) represents the input signal, and *y*(*k*) represents the output signal after whitening. The signal waveform is shown in Figure 11a and the spectrogram is shown in Figure 11b. They are both the results after the pre-whitening pretreatment. All the frequencies mentioned in this paper are normalized relative to the max value of the working band.

The spectra of the $p,v_{x},v_{y}$ and $v_{z}$ signals collected by each channel of the vector sensor contain a large number of line spectrum components. The harmonic line spectrum signal of the aerial target radiated noise can be clearly observed. The line spectrum of the vertical velocity $v_{z}$ is clearer than the other three signals ($p,v_{x},v_{y}$). Because the pressure (*p*) is omnidirectional, while the velocity ($v_{x},v_{y},v_{z}$) has the dipole directivity, the vertical velocity $v_{z}$ has vertical directivity, and its directivity is zero in the horizontal direction, so the noise in the horizontal plane has the less influence on the $v_{z}$ signal spectrum than the other three signal ($p,v_{x},v_{y}$) spectra. Furthermore, the traffic noise is the main source of the interference, and the traffic noise reaches in the horizontal direction, so the spectrum of $v_{z}$ has better effect. Using the $v_{z}$ spectrum to estimate the aerial target parameters is of great value [23].

In addition to the harmonic spectrum characteristics of the aerial target radiated noise, there are also harmonic interferences. The harmonic interferences caused by the observation platform are mainly caused by the existence of analog filter circuit electromagnetic effect. This part of harmonic interferences are power frequency (50 Hz) and its harmonic interference. Therefore, the algorithm which can eliminate the power frequency interference should also be added in the preprocessing process.

**Figure 12:**
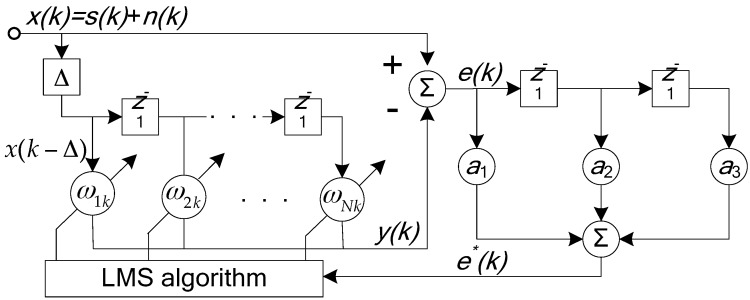
The schematic diagram of adaptive line spectrum enhancer (ALE).

The adaptive line spectrum enhancer (ALE) schematic block diagram is shown in Figure 12, $w_{1k}、w_{2k}\cdots w_{Nk}$ are the adaptive canceller’s weight coefficients; the input signal x(k) is a mixed signal of the noise n(k) and the continuous wave (CW) signal s(k); the delayed signal x(k−Δ) is regarded as the reference signal of the adaptive canceller, y(k) is the adaptive filter output signal. e(k) is called the residual signal. 

In order to strike a balance between the various performance requirements, the least mean square (LMS) algorithm is generally used in the adaptive line spectrum enhancer. The recursive formula is as follows:

(37) $$y(k)=\sum_{n=1}^{N}w_{n}(k)x(k-\Delta -n+1),e(k)=x(k)-y(k),w_{n}(k+1)=w_{n}(k)+\mu e(k)x(k-\Delta -n+1)$$

μ is a constant parameter whose value is 10^−5^.

**Figure 13:**
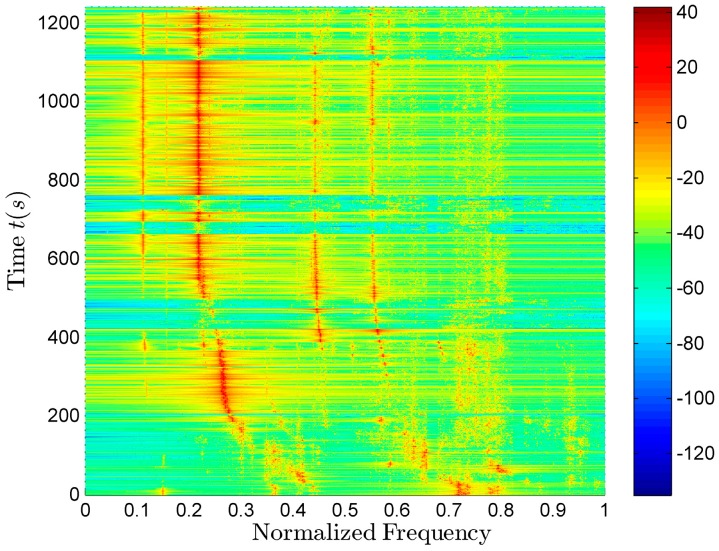
The adaptive line spectrum enhancement results of the $v_{z}$ signal.

The adaptive line spectrum enhancement results of the $v_{z}$ signal are shown in Figure 13.

### 4.2. Azimuth Estimation Results

**Figure 14:**
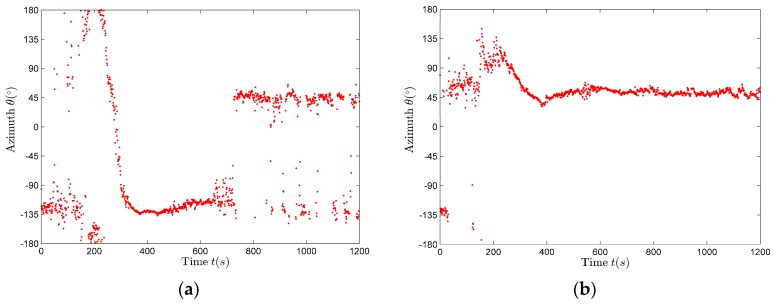
The azimuth estimation results. (**a**) Using the real part for calculation; (**b**) Using the imaginary part for calculation.

It can be seen from Figure 13 that in the band from 0.05 to 0.15, there are a large number of relatively continuous line spectra of great intensity. Therefore, for the signals in the band from 0.05 to 0.15, the azimuth estimation is performed using the frequency domain complex acoustic intensity method represented by (9). The spectrum integration length is 6 s, the step is 1 s. A cross-spectrum algorithm is applied using the fast Fourier transform (FFT) results. If we use the real part of the cross-spectrum results to estimate the azimuth, the results are shown in Figure 14a. The azimuth distribution is irregular and dispersive, and does not fit the trend of the actual movement. If we use the imaginary part of the cross-spectrum results to estimate the azimuth, the results are shown in Figure 14b. The azimuth distribution is similar to the actual movement trend. The target hovered around 50° before it moved off, and passed above the sensor at about 220 s. 

The traditional method is to estimate azimuth by calculating the ratio between the real part of $pv_{x}$ and the real part of $pv_{y}$. According to (35), in the very low frequency band, there is phase difference between the pressure and velocity of the actual field. Acoustic energy of the real component flows to the imaginary component, so the azimuth estimation results using the real component are likely to be of no value, but the azimuth estimation results using the imaginary part may be more desired, so when calculating the azimuth, the real part and imaginary part of the sound intensity flow need to be considered at the same time, in order to verify which algorithm is more effective.

By comparing Figure 14a with Figure 14b, using the imaginary part of sound intensity flow to calculate the azimuth is more effective in this sea experiment data processing.

### 4.3. Post-Processing Methods

#### 4.3.1. Moving Window-Weighted Median Filtering Method

The measured data is $x_{k}$ (k=1,2,⋯,N), the random error is subject to the Gaussian distribution. The data in the *m*-th window are called the *m*-th frame data, and there are *n* data $x_{m-n+1},x_{m-n+2},\cdots ,x_{m}$ in one frame. The horizontal vector composed of these *n* data is called the *m*-th frame vector. Their median is [26]:

(38) $$median(x_{m})=\{\begin{array}{cc} {}[x_{m}(\frac{n}{2})+x_{m}(\frac{n}{2}+1)]/2 & n=2k\\ x_{m}(\frac{n+1}{2}) & n=2k+1 \end{array}$$

the residual difference $r_{m}$ is the difference between the input value and the median of the output. $r_{m}=\;x_{m}-median(x_{m})$. The random error is subject to the Gaussian distribution, so the residual is also subject to the Gaussian distribution. There is an empirical coefficient of 1.3 between the residual’s standard deviation $\sigma_{m}$ and the mean value of the residual error’s absolute value (namely $mean(\left|r_{m}\right|)$). $\sigma_{m}=\;1.3\times mean(\left|r_{m}\right|)+\;10^{-12}$. The introduced constant coefficient in the formula is to prevent $\sigma_{m}$ equal to zero. Define a threshold $th_{m}$, $th_{m}=\;4.6*\sigma_{m}$, based on the variance of the residual. Then define a weight coefficient $w_{m}$:

(39) $$w_{m}=\{\begin{array}{cc} {\left\{1-{\left[r_{m}(i)/th_{m}\right]}^{2}\right\}}^{2} & i=1,2,\cdots ,n\left|r_{m}(i)\right|\leq th_{m}\\ 0 & i=1,2,\cdots ,n\left|r_{m}(i)\right|>th_{m} \end{array}$$

The smaller the absolute value of the residual is, the larger $w_{m}$ is and the closer $w_{m}$ is to 1. When the absolute value of the residual is greater than or equal to 4.6 times of the standard deviation, it means that the residual falls outside the range from −4.6$\sigma_{m}$ to +4.6$\sigma_{m}$. When there is no wild point, the probability that the data fall outside the range from −4.6$\sigma_{m}$ to +4.6$\sigma_{m}$ is 0.000002.

The data after the window-weighted smoothing are recorded as $z_{m}(j),j=1,2,\cdots ,N$:

(40) $$z_{m}(j)=\frac{\sum_{i=m-n+1}^{m}x_{n}(i)\cdot w_{m}(i)}{\sum_{i=m-n+1}^{m}w_{m}(i)}$$

#### 4.3.2. Forward and Backward Double α Filtering Method

The output of the input sequence X(k) after forward α filtering is ${\hat{X}}_{1}(k)$. The specific algorithm is as follows:

(41) $$\{\begin{array}{ll} {\hat{X}}_{1}(k)=X(k) & k=1\\ {\hat{X}}_{1}(k)={\hat{X}}_{1}(k-1)+\alpha \cdot [X(k)-{\hat{X}}_{1}(k-1)] & k=2,3,4,\ldots N \end{array}$$

The output of the input sequence X(k) after backward α filtering is ${\hat{X}}_{2}(k)$. The specific algorithm is as follows:

(42) $$\{\begin{array}{ll} {\hat{X}}_{2}(k)=X(k) & k=1\\ {\hat{X}}_{2}(k)={\hat{X}}_{2}(k-1)+\alpha \cdot [X(k)-{\hat{X}}_{2}(k-1)] & k=N-1,N-2,\ldots 1 \end{array}$$

The forward double α filter output is:

(43) $$\hat{X}(k)=\frac{1}{2}({\hat{X}}_{1}(k)+{\hat{X}}_{2}(k))$$

α is the integral time constant in (41) and (42). In addition to the selection of α value, the forward double α filter smoothing effect is closely related with the selection of the initial value, so we should select datum in the stable time period as the filter starting point.

As shown in Figure 14b, the azimuth estimation results have a big variability relative to the true value, mainly in the first 100 s (during this period, the true value of the azimuth should decreases from about 75° to about 45° and then increases from about 45° to about 135° gradually), because the underwater observation platform is still moving. Therefore, these parts of the data are discarded during the parameter estimation, and the rest of the stable data observed by platform are used in the estimation. The combination of the moving window-weighted median filtering method and the forward double α filtering method is used to post-process the data, which can effectively remove the wild points. The smoothing effect of the second half of the filtering results is very good, but the smoothing effect of the first half of the filtering results is very poor, especially the smoothing effect of the initial part of the filtering results. Reversing the smoothed data $\hat{X}(k)$, namely $Y(k)=\hat{X}(N-k+1)$, and then using the combination of the moving window-weighted median filtering method and the forward double α filtering method to post-process the data Y(k) again, is called backward double α filtering method. At this time the first half of data can also be smoothed, so the whole segment of data are smoothed so that the smoothing results retain more useful data.

**Figure 15:**
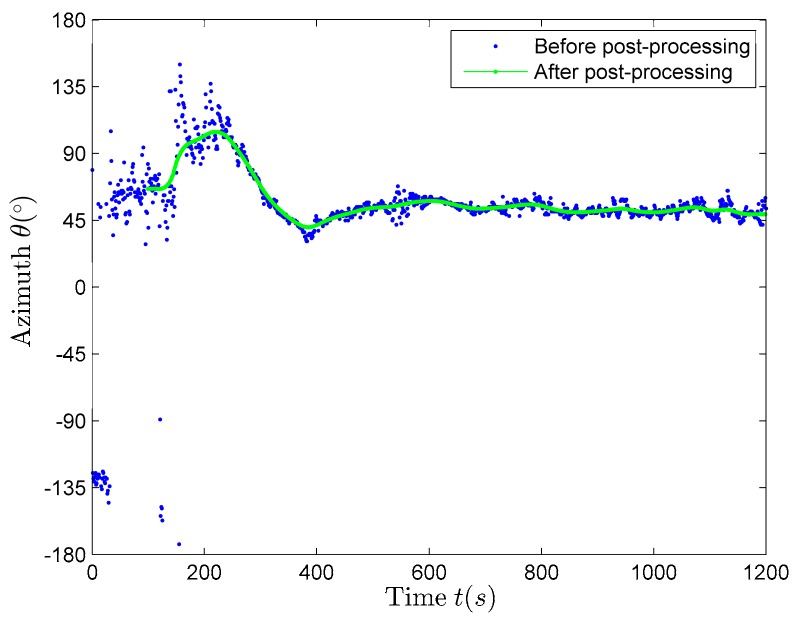
The estimation results of the azimuth before and after post-processing.

The comparison of the azimuth estimation results before and after post-processing is shown in Figure 15, where we can find that the whole segments of data are smoothed well. This article attempts to use the forward and backward two-direction two-times combined post-processing method to eliminate the wild points.

### 4.4. Frequency Estimation Results

FFT spectrum estimation results should be smoothed by the weighted window. Sliding window processing can smooth the spectrum estimation results, but the sample truncation leads to spectrum leakage, spectrum leakage is also an important interference in the line detection. Thus the sliding window form cannot be the rectangular window, and must be the weighted window for suppressing the spectrum leakage. The weighted window only modifies the front edge and the back edge compared with the rectangular window. The flat top of the weighted window should be as long as possible. Both of the front edge and the back edge of the weighted window should be cosine functions, tangent to the flat top and the zero line. The Hamming window just meets the above needs of the weighted window. So we use Hamming window as the weighted window. Its window length is 3 s, and its step is 1 s.

We use the Hamming window as the weighted window for spectrum smoothing. We can use the smoothing results in the cross-spectrum azimuth estimation. Hamming window form:

(44) $$w(n)=\left[0.54-0.46\cos(\frac{2\pi n}{N-1})\right]R_{N}(n),R_{N}(n)=\{\begin{array}{cc} 1 & 0\leq n\leq N-1\\ 0 & else \end{array}$$

**Figure 16:**
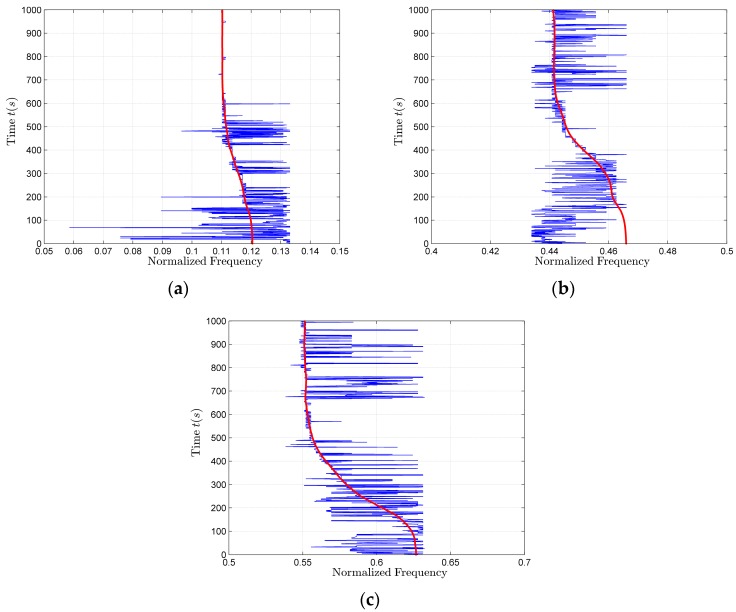
The spectra and frequency sequences extraction results. (**a**) In the band from 0.05 to 0.15; (**b**) In the band from 0.4 to 0.5; (**c**) In the band from 0.5 to 0.7.

It can be seen from Figure 13 that the line spectrum signals in the 0.05–0.15, 0.4–0.5 and 0.5–0.7 bands are clear. The signal spectra (blue solid line) and the extracted frequency sequences (red dot) in the three bands are shown in Figure 16a–c, respectively.

Signals in the 0.05–0.15, 0.4–0.5, 0.5–0.7 bands are used for parameter estimation. Through the least squares fitting of the three-band frequency sequences, we can get that the source frequency estimation results in the 0.05–0.15, 0.4–0.5, 0.5–0.7 bands are 0.1140, 0.4680 and 0.5860, respectively, while, the true values of the source frequency in the above three bands are 0.1167, 0.4667 and 0.5833. The source frequency estimation result (0.1140) in the 0.05–0.15 band, in fact, is just the base frequency estimation result. By averaging the fitting results of the three-band frequency sequences within the first 500 s, we can get a better base frequency estimation result (${\hat{f}}_{01}=0.1168$), because the true value of the base frequency is 0.1167. The base frequency estimation result, obtained by using harmonic clusters enhance estimation algorithm, is ${\hat{f}}_{01}=0.11$. It can be seen from the extracted frequency sequence shown in Figure 16a that the estimation result ${\hat{f}}_{01}=0.11$ is clearly incorrect, ${\hat{f}}_{01}=0.11$ is the frequency value when the Doppler frequency offset is at the maximum position. The reason of poor estimation effect is that the harmonic clusters enhance algorithm will do data matching during the entire time period, and in the whole time, the proportion of the frequency close to the maximum Doppler frequency is large, resulting that the frequency at the maximum of the Doppler frequency offset is wrongly regarded as the base frequency in the harmonic cluster algorithm. Therefore, in this paper, we propose a forward and backward two-direction double α filtering method to extract longer frequency sequence. The time period, during which the proportion of the frequencies near the maximum Doppler frequency is moderate, is taken as the appropriate time period for the base frequency estimation, namely 1–500 s. At this time, the estimation result ${\hat{f}}_{01}=0.1168$ is obviously superior to the result obtained by using the harmonic cluster line enhancer.

### 4.5. Parameter Estimation Results in the Horizontal Direction

**Figure 17:**
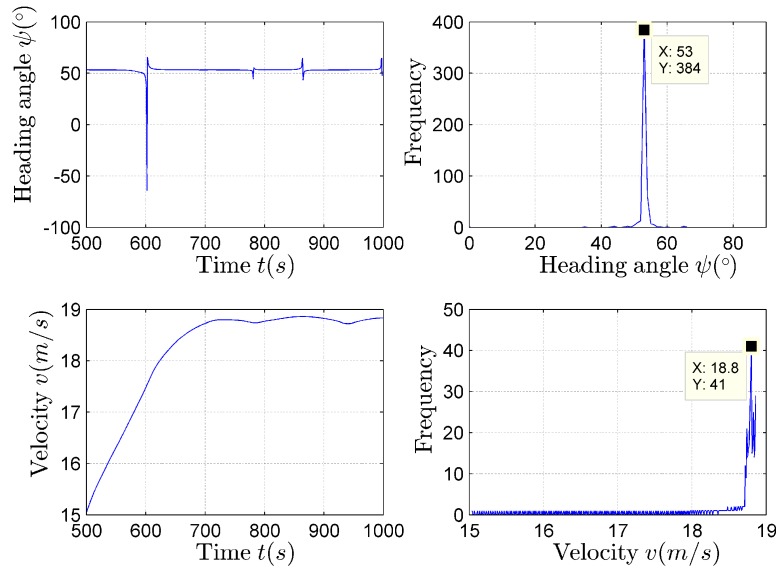
The heading angle and velocity estimation results.

The estimation results of the heading angle and the target velocity using (19) and (20) are presented in Figure 17.

The statistical analysis results of the heading angle and the target velocity are presented as the histogram in the paper, as shown in Figure 17. According to Figure 17, the parameter estimation values corresponding to the maximum in each histogram are the ideal heading angle and target velocity estimation results. 

**Figure 18:**
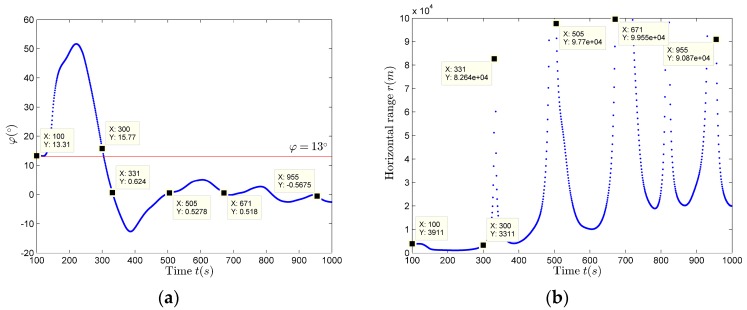
The horizontal distance Estimation Results if *p* = 900 m. (**a**) φ estimation results; (**b**) Horizontal distance estimation results.

It can be seen from Figure 18a,b that, when $\varphi =\theta -\psi \approx 0^{\circ }$, the horizontal distance estimation results carried out using (22) are discontinuous and inaccurate, and there are a large number of miscalculate maximum values. It can also be found that, during the time period 100–300 s, $\varphi >10^{\circ }$, the horizontal distance estimation results are relatively stable. Next, the *p* is scanned with the range of *p_match_* = 1–5000 m, and $\hat{v}$ is evaluated using the improved algorithm shown by (24). Each of the estimated ${\hat{v}}_{match}$ expression corresponding to $p_{match}$ is: ${\hat{v}}_{match}=\;\bar{\hat{v}(100:300)}$. The $p_{match}$ corresponding to the ${\hat{v}}_{match}$ closest to the velocity estimation result obtained by using (20) is the most desired closest distance estimation result ${\hat{p}}_{match}$. After scanning, the best closest distance estimation result is ${\hat{p}}_{match}$ = 744 m, at the same time ${\hat{v}}_{match}=\;18.8116\;m/s$.

**Figure 19:**
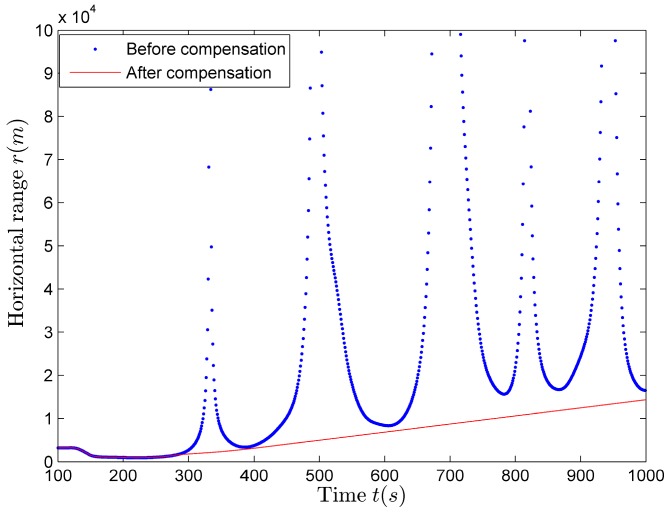
The estimation results of the horizontal distance before and after compensation.

If we obtain the closest distance estimation results, we can estimate the horizontal distance using (22). Compared with the horizontal distance estimation results shown in Figure 18b, there is only a difference in amplitude, the trend is still the same, that is, there is still the situation of discontinuity and inaccuracy. In this case, the compensation of the horizontal distance estimation results should be made using (25), so as to obtain better estimation results. The horizontal distance compensation results are shown in Figure 19 (red solid line), the estimation results before compensation are also shown in Figure 19 (blue dot).

The parameter estimation results of the target in the horizontal direction are listed in Table 3.

### 4.6. Parameter Estimation Results in the Vertical Direction

**Figure 20:**
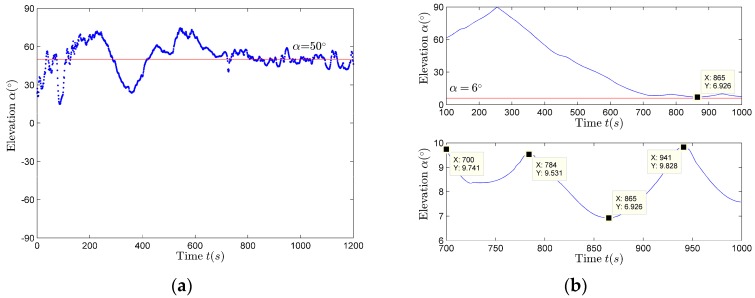
The elevation estimation results. (**a**) Elevation estimation results using the vertical sound intensity flow method; (**b**) Artwork and magnification of the elevation estimation results using the frequency sequence extraction method.

With the increase of the source movement time, r>>H, that is $\alpha \to 0^{\circ }$. Obviously the elevation estimation results using (27) and (28) do not satisfy this condition, as shown in Figure 20a. However, the elevation estimation results using (29) satisfy this condition, as shown in Figure 20b. Therefore, it is better to use (29) to evaluate the elevation. The reason why using (27) and (28) to evaluate the elevation angle is not good, may be linked to the limited accuracy of the data collected by three-dimensional vector sensor in the $v_{z}$ direction or the limited accuracy of the elevation estimation or the higher angle estimation pre-processing requirements. In Figure 20b, the elevation data during 700–1000 s are relatively stable, and satisfy $\alpha >6^{\circ }$, so this time period is selected for height estimation.

**Figure 21:**
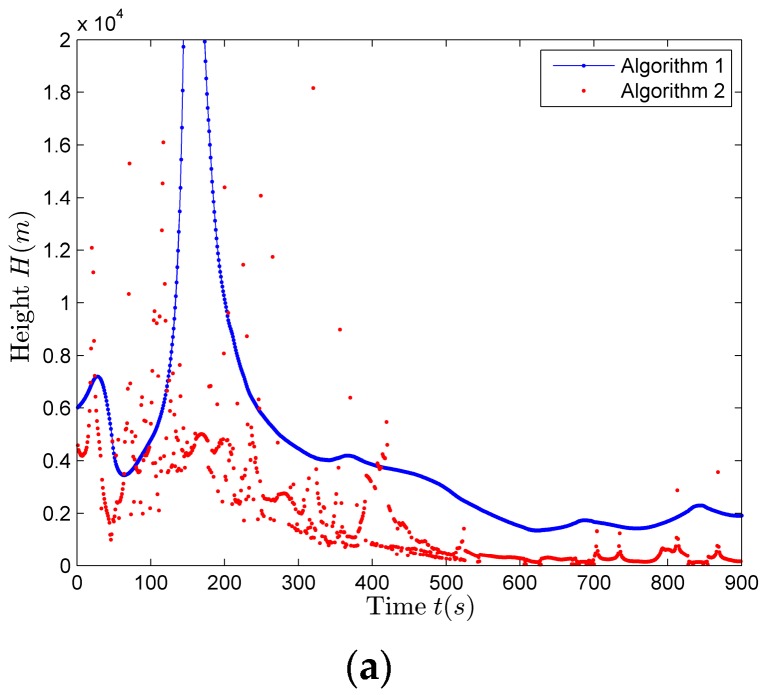

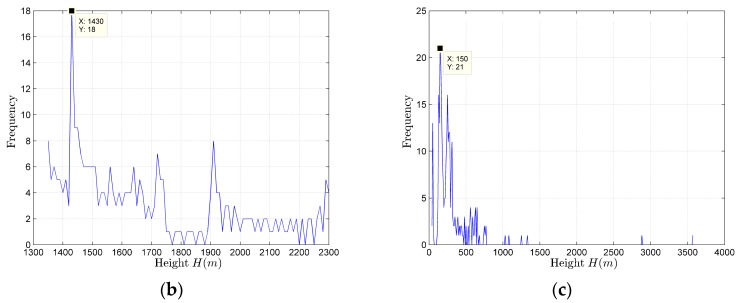
The height estimation results of the aerial target. (**a**) The height estimation results of the two algorithms; (**b**) The histogram statistical results of Algorithm 1; (**c**) The histogram statistical results of Algorithm 2.

Figure 21a compares the height estimation results considering Algorithm 1, using (31), with the Algorithm 2, using (34). It can be seen that height estimation results of the Algorithm 2 are more concentrated and more stable. The histogram statistical results of the two algorithms are shown in Figure 21b,c, respectively. Comparing Figure 21b with Figure 21c, Algorithm 2 has better accuracy and more robust than the Algorithm 1, for the reason that Algorithm 2 has lower requirements on the accuracy of φ. The final height *H* (from aerial target to receiver) estimation result is 150 m, so the aerial target height is 125 m (the reference surface: the sea surface).

## 5. Conclusions

In this paper, based on the existing aerial target localization methods, we propose an improved method so as to solve the problem that the parameter estimation results obtained by using the existing methods are less-than-ideal when θ≈ψ. The existing methods have good effect in estimating parameters in the simulation and actual data processing only when θ≠ψ. When θ≈ψ, although the simulation results of the existing localization methods are good, the high accuracy requirements of the angle estimation cannot be met in the actual data processing, so that the closest distance is difficult to estimate accurately whence the horizontal distance estimation results are unsatisfactory, so the accuracies of the measurement results, based on the elevation and the horizontal distance estimation, are poor. In this paper, the improved method is proposed to make the aerial target motion parameter estimation results more robust, and to reduce the accuracy requirements of the angle estimation results. How to select the appropriate time period for the parameter estimation is described in detail. At this time, we realize the more accurate parameter estimation in the situation that θ≈ψ. The more accurate parameters estimation results provide a good foundation for our next work. The idea of using forward and backward two-direction double α filtering method in the post-processing is also proposed, which can smooth the data better and retain more data information. This filtering method can also be used in the other application domains. We also propose two height measurement methods that can be used in the actual aerial target sea experiment data processing. Through the parameter estimation algorithm, the horizontal distance and vertical height of the aerial target are well estimated, so as to realize the three-dimensional localization of the aerial target, which effectively improves the maneuverability of the underwater platform.

## Figures and Tables

**Table 1:** The simulation parameters.

Parameters	Value	Parameters	Value
Source base frequency $f_{01}$	40 Hz	Integration time length	2 s
Heading angle ψ	10°	Sampling frequency $f_{s}$	1 kHz
Velocity v	20 m/s	Target Height *H*	200 m
Closest distance p	700 m	Sound velocity in the air $c_{1}$	340 m/s
Initial distance $r_{0}$	1200 m	Sound velocity in the water $c_{2}$	1500 m/s
Signal Noise Ratio (*SNR*)	10 dB	Signal total duration *T*	200 s

**Table 2:** The parameter estimation results in the horizontal direction.

Parameters	Theoretical Value	Estimation Value
Source base frequency $f_{01}$	40 Hz	40.3 Hz
Heading angle ψ	10°	8°
Velocity v	20 m/s	20.4 m/s
Closest distance p	700 m	880 m

**Table 3:** The parameter estimation results in the horizontal direction.

Motion Parameters	Estimation Results
Source frequency ${\hat{f}}_{01}$	0.1167
Heading angle $\hat{\psi }$	53.0°
Velocity $\hat{v}$	18.8 m/s
Closest distance $\hat{p}$	744 m
